# Mapping therapy-responsive immune ecotypes in clear cell renal cell carcinoma through integrative omics

**DOI:** 10.3389/fimmu.2026.1893607

**Published:** 2026-07-15

**Authors:** Zheng Zhang, Lijun He, Bin Chen, Huanle Zhang

**Affiliations:** 1 Department of Radiotherapy, Suzhou Ninth People's Hospital, Suzhou, China; 2 Department of Ultrasound, The Second Affiliated Hospital of Army Medical University, Chongqing, China

**Keywords:** clear cell renal cell carcinoma, immune checkpoint blockade, immune ecotypes, immunotherapy response, integrative omics, spatial transcriptomics, therapy resistance, tumor microenvironment

## Abstract

Clear cell renal cell carcinoma (ccRCC) is a kidney cancer in which immune activity is closely intertwined with von Hippel-Lindau (VHL) loss, hypoxia-inducible factor (HIF) signaling, angiogenesis, hypoxia, and metabolic adaptation. Although immune checkpoint inhibitor (ICI)-based regimens have changed the treatment landscape of advanced ccRCC, only a subset of patients achieve durable benefit. Commonly used biomarkers, such as programmed death-ligand 1 (PD-L1) expression, tumor mutation burden (TMB), and broad inflammatory gene signatures, have not been sufficient to explain this variation or to guide routine treatment selection. One reason is that immune infiltration in ccRCC is not synonymous with effective antitumor immunity. A tumor rich in CD8+ T cells may still be resistant if these cells are exhausted, metabolically restricted, spatially separated from tumor nests, or surrounded by suppressive myeloid, stromal, and vascular programs. Therefore, the key issue is not simply whether a tumor is immunologically “hot” or “cold,” but which part of the antitumor response has failed. In this Mini Review, we discuss ccRCC immunotherapy response from an immune-ecological perspective. We focus on several treatment-relevant immune states, including T-cell-inflamed but dysfunctional tumors, myeloid-dominant suppressive tumors, angiogenesis- and hypoxia-skewed tumors, and immune-excluded tumors. We also consider how bulk transcriptomics, single-cell and spatial profiling, T-cell receptor sequencing, proteomics, metabolomics, and longitudinal liquid biopsy may help define these ecotypes and capture treatment-induced remodeling. This perspective may support more refined patient stratification and more mechanism-matched immunotherapy strategies in ccRCC.

## 1 Introduction

Clear cell renal cell carcinoma (ccRCC) is the most common histological subtype of kidney cancer, but it differs biologically from many other epithelial tumors. In most sporadic cases, its development is closely linked to inactivation of the von Hippel–Lindau (VHL) tumor suppressor gene. When VHL function is lost, hypoxia-inducible factors, especially hypoxia-inducible factor 2 alpha (HIF-2α), can remain active even in the absence of true hypoxia. This abnormal signaling promotes vascular endothelial growth factor (VEGF)-driven angiogenesis, lipid and glycogen accumulation, and the development of a dense but disorganized vascular network. As a result, ccRCC grows within a microenvironment where vascular remodeling, metabolic stress, and immune regulation are tightly connected (1, 2).

Treatment for advanced ccRCC has changed markedly over the past two decades. High-dose cytokine therapy and sequential VEGF pathway inhibition have largely given way to immune checkpoint inhibitor (ICI)-based combinations. These include dual checkpoint blockade with nivolumab plus ipilimumab in CheckMate 214 and PD-1 blockade combined with VEGF receptor tyrosine kinase inhibition, such as pembrolizumab plus axitinib in KEYNOTE-426 (3, 4). These regimens have improved survival for many patients, but the clinical benefit remains uneven. Some patients achieve durable disease control, whereas others progress early despite receiving combinations that are biologically well justified. This contrast has shifted attention from the question of whether immunotherapy can work in ccRCC to the more difficult question of why similar regimens produce such different outcomes.

One limitation is that the usual distinction between “hot” and “cold” tumors does not fully fit ccRCC. In many tumor types, abundant CD8+ T-cell infiltration is taken as evidence of pre-existing antitumor immunity. In ccRCC, the relationship is less straightforward. High CD8+ T-cell infiltration in nephrectomy specimens has been associated with poorer survival, suggesting that immune abundance alone does not indicate immune effectiveness (5). T cells may be present but exhausted, metabolically impaired, spatially separated from tumor nests, or surrounded by suppressive myeloid, stromal, and vascular programs. Current biomarkers, including PD-L1 staining, International Metastatic Renal Cell Carcinoma Database Consortium (IMDC) risk classification, tumor mutation burden, and broad gene-expression signatures, each capture part of this biology, but none is sufficient on its own for reliable treatment selection (6). These limitations suggest that ccRCC should be viewed not only through fixed biomarker categories, but also as a set of changing immune states.

An immune ecology framework is useful in this context because it focuses on relationships rather than isolated markers. It asks how tumor cells, stromal cells, blood vessels, and immune populations are arranged, which cell types share the same niche, which ligand-receptor signals are active, and whether the vascular and metabolic environment supports or restricts antitumor immunity (7). Multi-omics approaches can help connect these different layers, including bulk transcriptional programs, single-cell states, spatial architecture, somatic alterations, proteomic networks, and longitudinal clinical outcomes. In this review, we summarize treatment-relevant immune ecotypes in ccRCC, discuss how multi-omics technologies may define and track these states, and consider how this perspective could support more precise biomarker development and mechanism-matched combination therapy.

Importantly, an ecological view does not replace established clinical and molecular biomarkers. Instead, it provides a broader framework for interpreting why the same biomarker may have different meanings in different tumor contexts. PD-L1, IMDC risk group, and TMB each capture a real but partial dimension of ccRCC biology, and their individual limitations are well documented: PD-L1 immunohistochemistry is confounded by antibody clone, scoring cut-off, and spatial and temporal heterogeneity, and has not reliably predicted ICI benefit in ccRCC; the IMDC model was developed as a clinical prognostic tool in the targeted-therapy era and captures host and disease burden rather than tumor immunobiology; and TMB, a robust predictor in several other tumor types, is characteristically low in ccRCC and correlates poorly with response. These markers also conflict in practice, so that a favorable-risk, PD-L1-negative, TMB-low tumor may still respond durably to ICI-based therapy, while an apparently “inflamed” PD-L1-positive tumor may progress. An immune-ecotype framework offers a way to reconcile such discordance by treating these markers as inputs to be interpreted within a spatial and functional context, rather than as independent yes/no predictors. In this sense, the most realistic near-term clinical value may lie in composite models that nest established biomarkers within ecotype-level information—for example, combining PD-L1 and IMDC group with the presence of tertiary lymphoid structures or a myeloid-suppression signature—an integration whose predictive gain over individual markers still requires prospective validation (6, 7).

## 2 Immune ecotypes and therapeutic pressure in ccRCC

**Figure 1:**
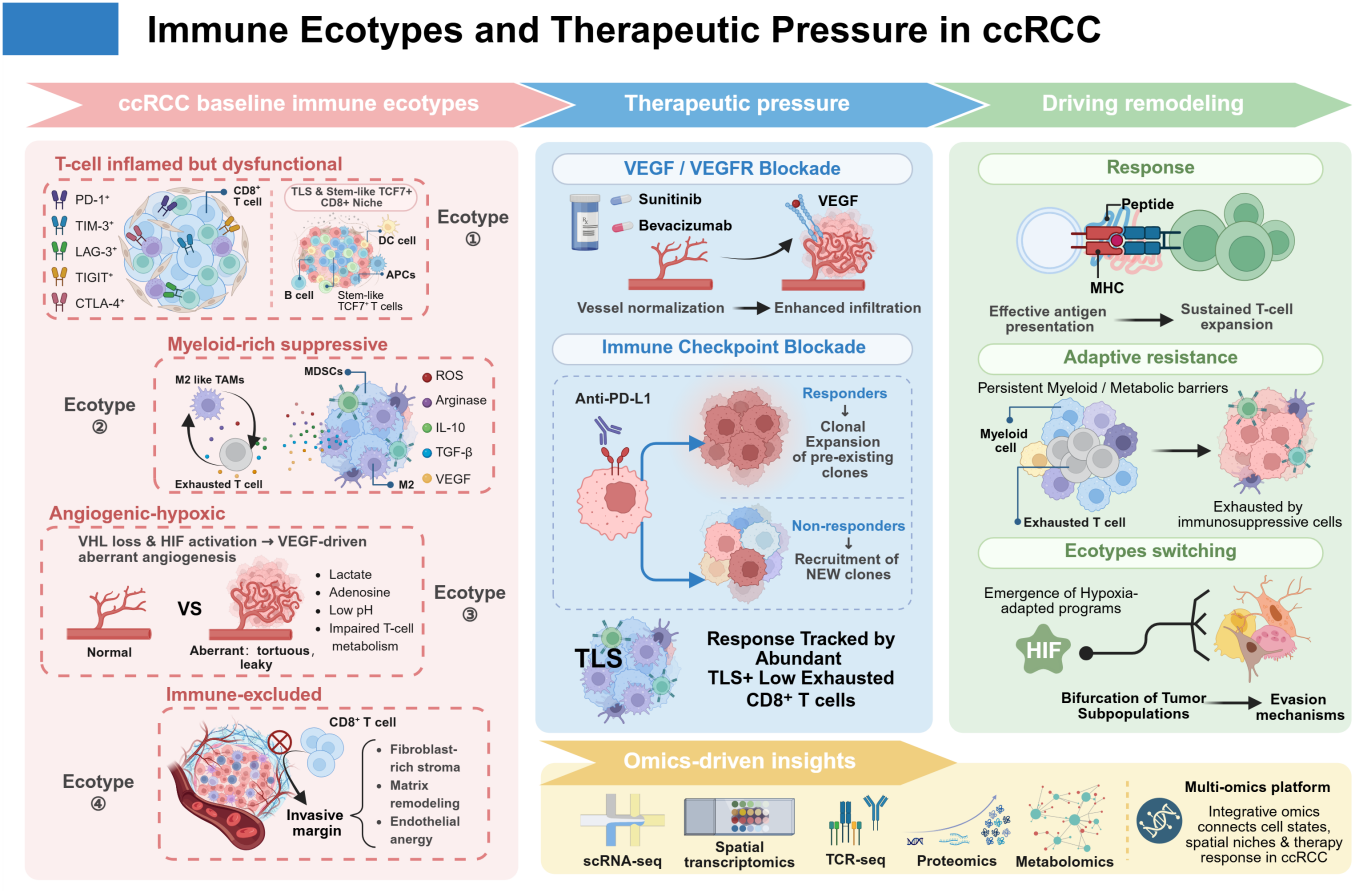
Immune ecotypes and therapeutic pressure in clear cell renal cell carcinoma.

Immune ecotypes in ccRCC are better regarded as recurring patterns of interaction among tumor cells, immune cells, stromal components, and vascular structures, rather than as fixed molecular subtypes. This distinction is important because a single tumor can contain more than one local immune state, and different metastatic lesions from the same patient may differ markedly in immune composition, as shown by multiregional sampling studies (8). Still, these recurring patterns are useful because they provide a way to connect mechanism with treatment response. In ccRCC, several treatment-relevant immune states are particularly important: T-cell inflammation with functional impairment, myeloid-dominant suppression, angiogenesis- and hypoxia-related immune restriction, and spatial immune exclusion (9) (**Figure 1**).

### 2.1 T-cell-inflamed but dysfunctional ecotypes

A T-cell-inflamed tumor is often assumed to be more responsive to immune checkpoint inhibition. In ccRCC, this assumption is not always valid. Single-cell studies have shown that many tumors contain abundant CD8^+^ T cells expressing cytotoxic molecules, including granzymes and perforin. However, these cells often also express inhibitory receptors such as programmed cell death protein 1 (PD-1), T-cell immunoglobulin and mucin-domain containing-3 (TIM-3), lymphocyte-activation gene 3 (LAG-3), T-cell immunoreceptor with immunoglobulin and ITIM domains (TIGIT), and cytotoxic T-lymphocyte-associated protein 4 (CTLA-4). This phenotype is consistent with chronic antigen exposure and progressive T-cell exhaustion (10, 11). The multiregional study by Krishna and colleagues made this point more clearly: response to PD-1 blockade was not explained simply by the number of infiltrating T cells, but was associated with tertiary lymphoid structures and a lower proportion of tissue-resident exhausted CD8^+^ T cells (8). Therefore, dense T-cell infiltration in ccRCC may sometimes reflect ineffective or chronically strained immunity rather than a tumor that is ready to respond to immunotherapy (5).

In this ecotype, the state and location of T cells may be more informative than their abundance. Kissick and colleagues identified an antigen-presenting cell niche in kidney tumors that supports TCF7^+^ stem-like CD8^+^ T cells. These progenitor-like cells help maintain terminally differentiated PD-1^+^TIM-3^+^ effector cells, whereas tumors lacking this niche appear more likely to progress (12). Findings from integrated single-cell ribonucleic acid sequencing (scRNA-seq) and T-cell receptor (TCR) sequencing point in the same direction. In responders, nivolumab tends to preserve and reactivate pre-existing expanded TCR clones. In non-responders, treatment is more often accompanied by recruitment of new clones that replace the original repertoire (13). This suggests that durable response depends not only on whether T cells are present, but also on whether an existing antigen-driven response can be sustained and restored. Regulatory T cells and dendritic cells add another layer to this balance. Regulatory T cells can suppress antitumor immunity through interleukin-10 (IL-10), transforming growth factor-beta (TGF-β), and competition for interleukin-2 (IL-2), whereas dendritic cells may support productive immunity through type I interferon signaling and co-stimulatory signals (14).

This inflammatory but dysfunctional state may help explain why dual checkpoint blockade benefits only some patients. CTLA-4 blockade can enhance early T-cell priming or reduce regulatory suppression, while PD-1 blockade can reinvigorate exhausted effector cells. These effects are unlikely to be sufficient, however, if the dominant barrier is poor antigen presentation, metabolic stress, or exclusion of T cells from tumor nests. In such settings, adding more immune stimulation may have limited antitumor effect and may mainly increase toxicity. The practical point is that a T-cell-rich ccRCC lesion should not automatically be considered an immunotherapy-sensitive lesion.

### 2.2 Myeloid-rich, angiogenic, and immune-excluded ecotypes

Another group of ccRCC immune states is shaped by myeloid enrichment, abnormal vasculature, hypoxia, and spatial immune exclusion. These features are best considered together because they often reinforce one another. Tumor-associated macrophages (TAMs), monocytes, myeloid-derived suppressor cells (MDSCs), and immature dendritic cells can limit antitumor immunity through several mechanisms, including arginase activity, reactive oxygen and nitrogen species, prostaglandins, indoleamine 2,3-dioxygenase, IL-10, TGF-β, VEGF, and checkpoint ligand expression (15). At the same time, a strong myeloid signal is not necessarily suppressive in every tumor. Macrophages in ccRCC are heterogeneous. Some show inflammatory or antigen-presenting features, whereas others are more closely linked to angiogenesis, tissue remodeling, and immune suppression. During disease progression, M2-like TAMs may form a reinforcing circuit with exhausted CD8^+^ T cells, contributing to poorer prognosis (10). This may explain why a high “myeloid” score in bulk transcriptomic data can have different meanings across tumors: in one context it may reflect immune activation, whereas in another it may mark a suppressive barrier (16).

Vascular and metabolic programs add another layer to this pattern. VHL loss and HIF activation drive VEGF-dependent angiogenesis, but the resulting vessels are often leaky, tortuous, and functionally inefficient. Rather than simply supporting tumor perfusion, this abnormal vasculature can impair T-cell trafficking, raise interstitial pressure, and maintain hypoxia (17). Hypoxia then reinforces immune suppression through HIF-dependent cytokine programs, adenosine accumulation, lactate production, extracellular acidosis, and impaired effector T-cell metabolism (18). The clinical importance of this axis was illustrated in IMmotion151. Unsupervised analysis of 823 tumors identified seven transcriptomic clusters that could be broadly grouped into angiogenic and proliferative/T-effector branches. Atezolizumab plus bevacizumab improved progression-free survival in tumors with high T-effector expression, whereas high angiogenic features predicted greater benefit from sunitinib (19). These transcriptomic branches, however, have not translated into reliable treatment-selection tools. In the phase III JAVELIN Renal 101 biomarker analysis, related angiogenesis and immune signatures showed treatment-dependent associations, but no tested signature provided a sufficiently robust, independently validated discriminator for routine selection between avelumab plus axitinib and sunitinib (20). Further uncertainty comes from BIONIKK and subsequent cross-trial transcriptomic analyses, in which angiogenic molecular subtypes showed substantial benefit from nivolumab plus ipilimumab rather than mapping cleanly to VEGF-targeted treatment (21, 22). Differences in assay platforms, signature definitions, cutoffs, treatment regimens, and sampling further limit cross-trial reproducibility. Thus, the IMmotion151 signatures should be considered hypothesis-generating rather than validated predictive biomarkers, reinforcing the need to integrate tumor, immune, stromal, and vascular features.

Immune exclusion represents a related but spatially distinct state. These tumors are not defined by a lack of immune cells, but by their mislocalization. T cells may accumulate at the invasive margin or around blood vessels, yet fail to enter tumor nests. Fibroblast-rich stroma, extracellular matrix remodeling, endothelial non-responsiveness, and TGF-β-associated programs can together create a physical and biochemical barrier to immune entry (23). In ccRCC, this form of exclusion often overlaps with angiogenic signaling and myeloid enrichment, generating layered resistance that may not be adequately addressed by VEGF blockade or immune checkpoint inhibition alone. A tumor may therefore appear inflamed in bulk transcriptomic data while the relevant immune cells remain spatially separated from malignant cells.

Finally, these ecotypes should be interpreted as dynamic treatment-responsive states rather than fixed pretreatment categories (**Figure 1**). VEGF receptor inhibitors may transiently normalize vessels and improve immune cell trafficking, but they may also select for hypoxia-adapted programs. Immune checkpoint inhibitors can expand or preserve tumor-reactive T-cell clones, but may leave myeloid, vascular, or metabolic barriers intact. Bi and colleagues demonstrated this remodeling directly: after ICI treatment, macrophages in responders shifted toward a pro-inflammatory state within an interferon-rich environment, while also upregulating immunosuppressive markers. Tumor cells themselves diversified along angiogenesis and immune escape programs, with associations involving PBRM1 status and survival (24). Thus, early changes in T-cell function, myeloid polarization, vascular normalization, and tumor-intrinsic escape programs may be more informative than any single baseline label (8, 24).

## 3 Integrative omics approaches for immune ecotype mapping

Mapping immune ecotypes is not simply a matter of counting immune cells. The aim is to reconstruct how tumor genotype, transcriptional state, immune composition, spatial organization, and therapeutic pressure interact within the same disease context (**Table 1**). No single platform can resolve all of these layers. Bulk ribonucleic acid sequencing (RNA-seq) provides reproducible measurements in large cohorts, but it compresses malignant, immune, endothelial, and stromal signals into averaged profiles. Single-cell and spatial methods recover cellular and tissue-level resolution, but they are more costly, usually involve smaller sample sizes, and are more sensitive to tissue handling and processing conditions. A useful ecotype map therefore depends less on any one technology than on the careful integration of complementary measurements.

**Table 1:** Complementary roles and interpretive pitfalls of omics platforms in ccRCC immune ecotype mapping.

Platform	What it resolves	Trade-off in ccRCC
Bulk RNA-seq	Population-level programs: angiogenesis, T-effector, myeloid, antigen presentation, stroma	Relatively scalable and reproducible across phase III cohorts, but compartment-specific signals are averaged; therefore a single score rarely has a single biological interpretation. In IMmotion151 (Motzer 2020), the angiogenesis signature tracked sunitinib benefit, whereas T-effector-high tumors tracked atezolizumab-bevacizumab benefit.
Single-cell RNA-seq	Per-cell states bulk cannot split: exhausted vs. proliferating CD8^+^ T cells, angiogenic vs. inflammatory TAMs	Shows ccRCC “hot” tumors are often dysfunctional rather than simply infiltrated (Braun 2021) — but with two to six patients per study, the catalogues are richer than they are generalizable and need a bulk cohort to anchor them.
scRNA-seq + TCR-seq	Transcriptional state tied to the clonal identity of the same T cell	Separates real clonal expansion from passive infiltration: in ADAPTeR (Au 2021) nivolumab response followed maintenance of pre-existing clones, not new ones — though the single-cell arm rested on two patients, leaning on the bulk-TCR cohort.
Spatial & multiplex imaging	Where cells sit: tumor–T-cell contact, immune niches, mature vs. immature TLS, stromal barriers	In ccRCC, position outranks abundance — mature TLS export IgG/IgA plasma cells that correlate with ICI response (Meylan 2022). But “TLS-positive” is not one thing; only mature TLS carry the signal, and maturity is easy to overcall on a single section.
Genomic/epigenomic	3p drivers (VHL, PBRM1, BAP1, SETD2, KDM5C) and the chromatin state opening or silencing immune genes	Links genotype to phenotype — SETD2 to an immune-desert subtype, BAP1 to an inflamed/interferon program (Beuselinck 2015; Camp 2026). Yet PBRM1 has flipped between “predicts response” and “no effect” across cohorts, hinting the unit is the ecotype, not the gene.
Proteomic/metabolic	Pathway activity (VEGF, interferon, mTOR, HIF) and constraints (hypoxia, lactate, adenosine, tryptophan)	Captures pathway activity that may not be inferred from mRNA alone and can help distinguish T-cell exclusion from metabolic impairment; however, this layer is difficult to spatially localize and is most informative when integrated with imaging.
Longitudinal/liquid biopsy	Change over time: on-treatment biopsy, ctDNA kinetics, circulating immune phenotype	Tests whether therapy remodeled the intended ecotype rather than merely describing baseline biology, but requires prospective, serial, standardized sampling that remains uncommon outside dedicated trials such as ADAPTeR.

(a) IMmotion151: Phase III bulk-transcriptomic analysis identified angiogenesis- and T-effector-associated programs; these signatures remain exploratory and are not validated for routine treatment selection. (b) ADAPTeR: Prospective paired sampling linked nivolumab response to maintenance and reactivation of pre-existing T-cell clones; interpretation is limited by the small single-cell cohort.

### 3.1 Bulk, single-cell, and spatial reconstruction

Bulk transcriptomics remains the main starting point for ccRCC biomarker studies. In large cohorts, it allows angiogenesis, T-effector activity, myeloid inflammation, antigen presentation, cell-cycle activity, and matrix-related programs to be compared with clinical outcomes. The IMmotion151 dataset is one of the clearest examples of how these transcriptional programs can be linked to treatment response (19). Even so, these signatures need careful interpretation. A high interferon-γ score, for example, may indicate productive immune activation, but it may also reflect chronic exhaustion or inflammatory stress after treatment. An angiogenesis score can suggest sensitivity to VEGF pathway inhibition, while at the same time describing a vascular context that may shape the response to immunotherapy.

Single-cell RNA sequencing helps address some of this ambiguity by separating malignant, endothelial, myeloid, T-cell, B-cell, and stromal compartments. ccRCC atlases have identified exhausted, proliferative, and exhausted-proliferative CD8^+^ T-cell subsets, regulatory T cells, inflammatory and angiogenic macrophage states, and dendritic and endothelial populations that cannot be clearly resolved from bulk data alone (25). When paired with T-cell receptor sequencing, single-cell analysis can also distinguish general lymphocyte infiltration from true clonal expansion. This is important for tracking whether treatment restores pre-existing antigen-driven clones or instead recruits new clones into the tumor (13). These studies have also brought attention to less abundant but potentially important populations, such as mature dendritic cells and tissue-resident memory-like T cells, whose effects on response may be larger than their frequency suggests.

Spatial transcriptomics, multiplex immunofluorescence, imaging mass cytometry, and digital pathology add another layer by preserving tissue architecture. They can show whether CD8^+^ T cells are in direct contact with tumor cells, whether dendritic cells form supportive immune niches, and whether stromal or endothelial barriers keep effector cells away from malignant nests (26). This is especially relevant in ccRCC, where immune cells may be abundant but poorly organized. Tertiary lymphoid structures (TLS) are a good example. Spatial studies in renal cell carcinoma have shown that mature TLS can support B-cell differentiation into immunoglobulin G (IgG)- and immunoglobulin A (IgA)-producing plasma cells, and that TLS density and plasma-cell output are associated with immunotherapy response and survival (27, 28). These findings suggest that treatment-responsive ecotypes are shaped not only by which cells are present, but also by how those cells are arranged within the tumor.

### 3.2 Genomic, epigenomic, proteomic, and metabolic integration

Tumor-intrinsic alterations also shape immune ecotypes. Beyond VHL, recurrent driver events in ccRCC are concentrated on chromosome 3p and include PBRM1, mutated in approximately 30%-40% of tumors, BAP1 and SETD2, each altered in approximately 10%-15%, as well as KDM5C and components of the mechanistic target of rapamycin (mTOR) pathway (29). These chromatin and signaling regulators can influence inflammatory output, deoxyribonucleic acid (DNA) damage responses, metabolism, and immune recognition. Yet their immunological effects are context-dependent rather than linear. For example, the Beuselinck classification links SETD2 mutations to immune-desert subtypes and BAP1 alterations to inflammatory subtypes, whereas single-cell epigenomic analysis connects BAP1 deficiency with a tumor-intrinsic interferon program that combines inflammatory and immune-evasive features (30, 31). Because the same mutation may produce different immune consequences depending on co-mutation patterns and microenvironmental context, integrated models are more informative than single-gene prediction. The inconsistent performance of PBRM1 status as an ICI biomarker across studies is better interpreted in this framework: PBRM1 may modulate therapy response through its interaction with broader ecological states rather than acting as an isolated predictive marker.

Epigenomic profiling can clarify whether immune-related loci are accessible or repressed, helping explain why one tumor expresses chemokines and antigen-presentation machinery whereas another remains immune excluded (31). Proteomic and phosphoproteomic data add another necessary layer because cytokine signaling and kinase networks, including VEGF, interferon-γ, Janus kinase–signal transducer and activator of transcription (JAK–STAT), phosphoinositide 3-kinase–protein kinase B–mTOR (PI3K–AKT–mTOR), and HIF pathways, are not always faithfully inferred from messenger RNA (mRNA) abundance (32). Metabolomics and metabolic imaging further identify local constraints on immune function, such as hypoxia, lactate accumulation, lipid remodeling, tryptophan catabolism, adenosine signaling, and nutrient competition (18). When combined with spatial mapping, these metabolic readouts may help distinguish visually similar ecotypes that require different therapeutic strategies: one in which T cells are excluded by vascular and stromal barriers, and another in which T cells have entered the tumor but remain metabolically impaired.

Longitudinal sampling is indispensable because a pretreatment biopsy captures only one moment in an evolving therapeutic process. Early on-treatment biopsies, circulating tumor DNA (ctDNA) dynamics, plasma proteomic profiles, cytokine measurements, immune-cell phenotyping, and radiomic features can help determine whether a treatment is actually reshaping the ecological program it was intended to target (24). The most informative future models will likely combine baseline tissue features, early pharmacodynamic readouts, and outcome data, rather than relying on any single pretreatment measurement.

## 4 Therapy-responsive ecotypes and clinical translation

The value of immune ecotype analysis ultimately depends on whether it can improve therapeutic matching rather than merely refine biological classification. In T-cell-inflamed but dysfunctional ecotypes, PD-1-based therapy remains biologically plausible, particularly when spatial profiling shows that effector T cells are positioned within tumor nests, antigen-presenting cell niches are preserved, and tertiary lymphoid structures are present. When regulatory T-cell activity, CTLA-4-dependent suppression, or insufficient priming appears to dominate, dual checkpoint blockade, as exemplified by the CheckMate 214 strategy, may be more rational than PD-1 blockade alone. However, this decision must be weighed against toxicity, comorbidity, and performance status, because immune escalation is unlikely to benefit patients whose dominant resistance mechanism lies outside the checkpoint axis (3). Conversely, tumors with inflammation accompanied by marked myeloid suppression may require strategies that remodel macrophage or monocyte programs rather than simply intensify checkpoint inhibition.

For angiogenesis- and hypoxia-dominant ecotypes, combining ICI with VEGF pathway inhibition has a strong biological rationale. Since KEYNOTE-426, the clinical success of PD-1 blockade plus VEGF receptor tyrosine kinase inhibitor (VEGFR-TKI) therapy has reinforced the idea that vascular targeting can do more than suppress angiogenesis: it has been proposed to improve vascular function, reduce suppressive myeloid recruitment, and create a transient window in which T-cell trafficking into tumor tissue becomes more permissive (4, 17). It should be emphasized, however, that this vascular-normalization model remains largely unproven in ccRCC itself, having been extrapolated chiefly from other tumor types and preclinical systems. A recent study by Vuong and colleagues, using a transgenic ccRCC model together with on-treatment single-cell and imaging mass cytometry cohorts, directly challenges this assumption: rather than normalizing vessels to enhance T-cell entry, response to VEGFR-TKI/anti-PD-1 was linked to treatment-induced hypoxic necrosis and the emergence of hypoxia-responsive secreted phosphoprotein 1-positive (SPP1+) macrophages, while pretreatment hypoxia predicted worse outcomes and prolonged TKI exposure aggravated metastasis (33). These findings indicate that how VEGFR-TKIs actually potentiate ICI in ccRCC is still largely unknown, reflecting our limited understanding of the coupled tumor–immune–stromal interactions that shape response. They also argue strongly for studies that resolve spatial architecture in paired samples both before and after treatment, rather than inferring mechanism from baseline tissue alone. Any vascular or immune remodeling is unlikely to be static. Excessive or prolonged anti-angiogenic pressure can deepen hypoxia, increase metabolic stress, and select for resistant cellular programs. Thus, dose, timing, and sequencing may be as important as drug selection itself.

HIF-2α inhibition provides a more direct way to target one of the central dependencies of ccRCC. Belzutifan, a first-in-class HIF-2α inhibitor (34), has been approved by the U.S. Food and Drug Administration for advanced renal cell carcinoma after prior PD-1 or PD-L1 blockade and VEGF tyrosine kinase inhibition. In LITESPARK-005, belzutifan improved progression-free survival compared with everolimus and produced a higher objective response rate in previously treated advanced ccRCC, 22.7% versus 3.5% (34). Because HIF-2α signaling affects angiogenesis, metabolism, and cytokine programs, blocking this pathway may also alter the immune environment of ccRCC. This idea is now being explored in trials such as LITESPARK-011, which evaluates belzutifan plus lenvatinib, and LITESPARK-012, which includes pembrolizumab-containing belzutifan combinations (35, 36).

Whether these combinations can consistently improve ICI sensitivity remains unclear. Their risks also need careful interpretation, because ecotype-specific toxicity has not been prospectively defined. Anemia and hypoxia are known adverse effects of HIF-2α inhibition (34, 37), and these toxicities may become more relevant when belzutifan is combined with agents that also affect vascular or metabolic function. In angiogenesis- and hypoxia-dominant tumors, sustained inhibition of both HIF-2α and VEGF signaling could, at least in theory, intensify metabolic stress or favor hypoxia-adapted resistance. In myeloid-dominant tumors, additional treatment may increase toxicity without addressing the main suppressive mechanism. These possibilities require prospective testing with paired baseline and on-treatment tissue, and they should not yet be used to guide routine treatment selection. A rational combination should demonstrate that the intended change occurs within patient tumors, rather than simply bringing together two active drugs. The same standard should apply to strategies targeting myeloid biology, adenosine signaling, metabolic constraints, or stromal barriers.

Clinical translation will also depend on assays that can be used in routine practice. Full multi-omics profiling is unlikely to be feasible for every patient. A more realistic approach is to use deep discovery datasets, including single-cell, spatial, genomic, proteomic, and metabolic measurements, to build reduced but interpretable clinical tools. These may include targeted RNA panels, multiplex immunohistochemistry, spatially informed digital pathology, plasma protein panels, or composite models that combine ctDNA and imaging features (27). The aim should not be another long biomarker list. Instead, these tools should help classify tumors into clinically meaningful resistance states, such as exhausted inflammatory, myeloid-enriched, angiogenesis-hypoxia-dominant, immune-excluded, metabolically constrained, or mixed transitional ecotypes.

Adaptive trial designs are well suited to this problem. Rather than testing fixed combinations in unselected populations, future trials could enrich for patients whose tumors show the target ecotype and then use early tissue or liquid-biopsy endpoints to determine whether the expected biological change has occurred. Paired biopsies, standardized specimen handling, harmonized analytical pipelines, and prespecified pharmacodynamic endpoints will be needed to limit retrospective overinterpretation. Ecotype-guided therapy should also support treatment de-escalation, not only intensification. For example, a patient whose tumor contains well-organized immune neighborhoods and mature tertiary lymphoid structures may not need the most toxic dual-immunotherapy regimen, whereas a patient with dominant vascular or stromal exclusion may benefit more from a sequential strategy matched to that barrier (21, 35). The broader goal is to move from empirical combination therapy toward treatment strategies in which the main barrier to durable antitumor immunity is identified, monitored, and therapeutically modified with acceptable toxicity (36, 38).

## 5 Discussion and future directions

Immune ecology is a useful framework for ccRCC because it treats the tumor as a changing ecosystem rather than a fixed molecular category. This is especially relevant in ccRCC, where immune infiltration, angiogenesis, hypoxia, and metabolic reprogramming are all prominent, but their meaning depends strongly on local context. Dense T-cell infiltration may indicate productive antitumor immunity, chronic exhaustion, or inflammation trapped within suppressive niches. Strong angiogenic signaling may predict sensitivity to VEGF pathway inhibition, but it may also reflect a vascular state that limits immune access. The value of integrative omics therefore lies not only in describing tumor features, but in connecting cellular state, tissue architecture, genotype, pathway activity, and treatment response into a model that can be interpreted clinically.

Several principles should guide the next phase of ecotype-oriented ccRCC research. First, immune ecotypes should be viewed as dynamic states. Therapy can remodel vasculature, expand or contract T-cell clones, repolarize macrophages, and alter metabolic constraints within a relatively short time. Second, more immune stimulation is not always better. If the dominant barrier is myeloid suppression, hypoxia, poor antigen presentation, or stromal exclusion, additional checkpoint blockade may add toxicity without restoring effective antitumor immunity. Third, discovery platforms must be translated into practical assays. A complex multi-omics signature will have limited clinical value unless it can be simplified into reproducible tests that work across institutions, tissue-processing conditions, and patient populations.

Future studies should place greater emphasis on spatially informed biomarkers, paired baseline and on-treatment biopsies, ctDNA dynamics, circulating immune profiles, and pharmacodynamic endpoints. These tools should be used to ask a specific question: did the intended biological change actually occur after treatment? Functional validation will be equally important. Patient-derived organoids, fresh tumor slices, ex vivo immune co-cultures, and humanized models can help determine whether a candidate combination remodels the relevant niche, rather than merely shifting an isolated marker. This distinction matters because a therapy may increase immune activation in bulk data but still fail if effector cells remain spatially excluded, metabolically impaired, or disconnected from antigen-presenting compartments.

To move this framework toward practice, several steps are needed. The field first needs a more consistent definition of ccRCC immune ecotypes, supported by a minimal set of markers and spatial features that can be reproduced across platforms and cohorts. Prospective biomarker-stratified or adaptive trials should then test whether patients can be enrolled according to a dominant ecotype and whether early on-treatment changes predict later clinical benefit. At the same time, multi-omics discovery should be deliberately reduced to deployable assays, such as targeted RNA or spatial panels, multiplex immunohistochemistry, ctDNA kinetics, and circulating immune phenotyping. Computational models that integrate histology, spatial omics, genomics, and longitudinal liquid-biopsy data may help assign ecotypes and predict their trajectories, but they will need external validation and careful attention to interpretability.

Important biological gaps also remain. Current ecotype models still underrepresent spatial and temporal heterogeneity across primary and metastatic sites, the contribution of B cells and tertiary lymphoid structures, the immunological effects of treatment sequencing, and the possibility that some patients may benefit from de-escalation rather than intensification. If these issues are addressed carefully, ecotype-guided therapy could help existing treatment classes, including ICI, VEGF pathway inhibitors, CTLA-4 blockade, HIF-2α inhibitors, and metabolism- or myeloid-directed strategies, to be used with greater precision. The future of ccRCC immunotherapy may therefore depend less on adding more agents to every regimen and more on identifying the dominant resistance barrier, testing whether it can be modified, and adapting treatment before resistant states become fixed.
